# Hierarchical Fusion Using Subsets of Multi-Features for Historical Arabic Manuscript Dating

**DOI:** 10.3390/jimaging8030060

**Published:** 2022-03-01

**Authors:** Kalthoum Adam, Somaya Al-Maadeed, Younes Akbari

**Affiliations:** Department of Computer Science and Engineering, Qatar University, Doha P.O. Box 2713, Qatar; s_alali@qu.edu.qa (S.A.-M.); akbari_younes@semnan.ac.ir (Y.A.)

**Keywords:** historical Arabic manuscript dating, handwriting style-based features, sparse representation-based features, deep features, hierarchical fusion

## Abstract

Automatic dating tools for historical documents can greatly assist paleographers and save them time and effort. This paper describes a novel method for estimating the date of historical Arabic documents that employs hierarchical fusions of multiple features. A set of traditional features and features extracted by a residual network (ResNet) are fused in a hierarchical approach using joint sparse representation. To address noise during the fusion process, a new approach based on subsets of multiple features is being considered. Following that, supervised and unsupervised classifiers are used for classification. We show that using hierarchical fusion based on subsets of multiple features in the KERTAS dataset can produce promising results and significantly improve the results.

## 1. Introduction

**Figure 1:**
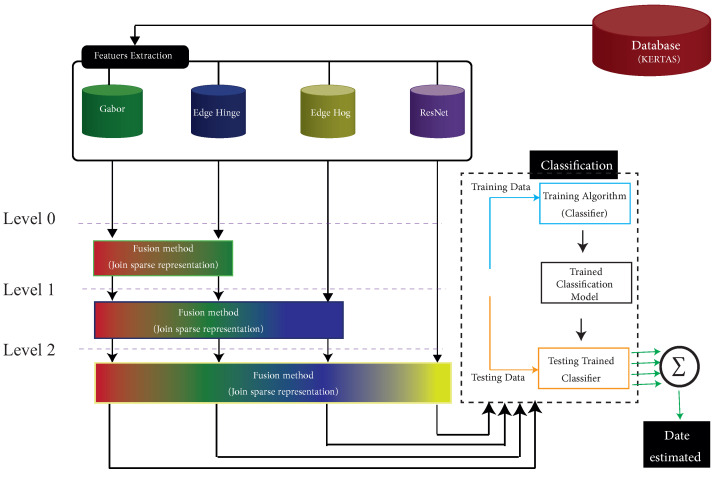
Overview of our proposed system.

Arabic manuscripts are an important part of Arab and Muslim heritage around the world. National libraries house hundreds of thousands of digital images; however, many documents do not expressly state when they were written. Dating historical documents will assist in linking them to an important event and determining their historical significance. Handwriting styles in Arabic evolved over time. Each Islamic century has its own set of writing scripts, giving the various writing styles distinct characteristics. Some writing styles evolved over centuries, retaining their general characteristics while also incorporating a new set of personalities. The degraded state of the historical documents, as well as the similarity of the writing styles, make it difficult to date the historical document. Several works on manuscript dating were performed, which we will look at in the following section. For instance, the System for Paleography Inspection (SPI) [1] was one of the earliest studies in the field of digital paleography. SPI for Latin documents breaks down the manuscripts into character images. Each new character image is tested against the exciting database using tangent distance and statistical-based algorithms. Despite extracting suitable features in the methods, these methods need to be improved. The combination of the features obtained promising results. However, these methods were traditional and only concatenated the features to feed into the classifier, e.g., [2]. This motivated us to use an effective combination method to fuse the feature. Although a new fusion method can demonstrate better results than a traditional one (with concatenating features), existing noise among the features can affect their accuracy. This paper presents a novel fusion approach by hierarchically considering subsets of the multifeatures. Selecting the subsets puts the approach open to the following research. However, we also explore some of the subsets in the study. A representation of the selected subsets with their corresponding levels included in the suggested approach is presented in Figure 1. Our approach is based on one of the popular fusion methods: the joint sparse representation. Fusion techniques in general, and particularly sparse-representation methods, struggle with unwanted noise when combining features, which affects the final output [3]. Therefore, to avoid that situation, we select subsets of the multifeatures to feed into the method hierarchically rather than simultaneously considering whole features. The main contributions of this paper are defined as follows:

- A novel approach for fusing multifeatures: the fusion approach is proposed describing a hierarchical structure based on subsets of the multifeatures;
- Exploring the type of subset selection: we try to cover some of the states of the selected features. A comparison of the states is reported in the paper;
- The first attempt: this work is the first attempt to conduct an investigation after introducing the KERTAS dataset to the best of the authors’ knowledge;
- Improved accuracy for historical manuscript dating: we show that the proposed techniques deliver better performance compared with that of the dating methods based on traditional feature fusions. Additionally, our approach obtains promising results compared to the same fusion method, while all features are considered simultaneously.

The rest of this study is organized as follows. Section 2 includes a literature review of related work, and Section 3 presents the suggested model. Experimental results are shown in Section 4, and Section 5 concludes this article.

## 2. Related Works

In this section, we first briefly mention some of the existing datasets used in historical documents studies. Later, we present an overview of the notable contributions to the automated analysis of handwriting for date estimation. Finally, we review some studies that research fusion methods, as well as the method types.

### 2.1. Datasets

In this subsection, we cover some of the historical manuscript datasets that are available online. The institute de recherche et dhistoire des textes (IRHT) has an online dataset that consists of more than 76,000 manuscripts in multiple languages, including but not limited to Latin, Hebrew, Greek, and Arabic  [4]. Other resources that have historical manuscripts’ images are [1,5,6,7]. More than 6000 documents from England and Wales of the Early England Data Set (DEEDS) are presented in [8]. The documents are dated from around the 11th to the 14th century. In [9], a new dataset was introduced. The MPS contained medieval charters that dated back to 1300–1550 CE. The 3267 charters in Medieval Paleographical Scale (MPS) were written in the ‘Medieval Dutch’ language. Sulaiman et al. in [10] proposed a dataset for degraded Arabic historical manuscripts dating to the Islamic and ancient Arabic eras. Meanwhile, Wahlberg et al. in [11] presented a dataset from the Swedish collection Svenskt Diplomatariums huvudkartotek (SDHK). The dataset was relatively large and consisted of more than 10,000 medieval charters from the Swedish collection.

The CLaMM [12], is a database for the Classification of Medieval Handwritings in Latin Scripts (CLaMM) competition at (ICDAR) 2017 conference. It consisted of 3540 images for style classification and manuscript dating dates from 500 CE to 1600 C.E. Another competition database is the Historical-WI database [13]. The database consists of 3600 colored and binarized images of handwritten historical documents written by 720 writers and five pages per writer.

The Dead Sea Scrolls (DSS) database was introduced in [14]. DDS contains 150 collections of Dead Sea Scrolls and consists of digitized manuscripts of 28 different spectral bands of light at a resolution of 1215 pixels per inch.

In [15], another multispectral database was presented. The MS-TEx database contained 240 multispectral images obtained from 30 historical handwritten letters dated from the 17th to the 20th centuries. The KERTAS dataset, which contains over 2000 images spanning 14 centuries, is the first attempt to create an Arabic manuscript dataset [16].

### 2.2. Automated Date Estimation from Handwriting

Analyzing digitized images of the historical manuscripts enabled automated dating and classifying of manuscripts. Current research in the field of digital paleography uses visual descriptors extracted from digitized images. Classification methods are used for age estimation based on these descriptors. While many of these methods rely on the content of manuscripts only, some methods propose using content-independent techniques. Overall, these methods can be classified into two categories: traditional and deep learning approaches.

Several studies proposed different automated date estimation techniques using MPS database, such as [9,17,18]. In [9], authors estimated the date of the historical documents by using a regression method that employed both local and global level features. The method used Hinge and Fraglets features.

He et al. in [18] presented a trained codebook method by combining both local contour fragment (kCF) and stroke fragment (kSF) features to estimate the age of a historical document.

A clustering algorithm to relate the low-level visual descriptors of the historical document to their labels in the MPS database was proposed in [17]. The method showed correlations between image descriptors and labels.

Based on shape statistics, Wahlberg et al. in [19] presented automated dating techniques for unbinarized gray images for the database. The proposed techniques were tested on the “Svenskt diplomatariums huvudkartotek” collection, which included scanned images of medieval charters kept in the Swedish national archive. In [20], authors employed convolutional neural networks (CNN) to predict the date of printed documents from the Google books corpus [21]. Hamid et al. in [2] suggested that using a number of combined features would provide better performance over using individual ones. The authors employed a combination of Gabor filters, Uniform Local Binary Patterns, and Histogram of Local Binary Patterns. In [22], authors presented a deep-learning-based approach using transfer learning on pretrained Convolutional Neural Network (CNN) models. Studer et al. in [23] presented a historical document dating technique using Transfer learning of pretrained neural networks on the ImageNet database as a part of diverse comprehensive research using the databases in [12,13,24,25].

One of the recent works in dating historical documents was conducted by Rahiche et al. in [15], who introduced a content-independent technique based on the optical properties of historical documents, such as discoloration and the changes in writing materials. The proposed method captures temporal information from iron-gall ink using the multispectral image technique combined with the kernel discriminant learning for an ordinal regression (KDLOR) classification approach. In another recent work [26], authors proposed using a grapheme-based method with the self-organizing time map (SOTM) as a codebook for dating the Dead Sea Scrolls collection.

### 2.3. Fusion Methods

The aim of the multifeature approach, is to reveal and relate the correlation of features across different views. Approaches to address this issue (similarity across features) can categorize into three groups of multikernel learning [27,28], subspace learning [29,30], and sparse representation [31,32]. Since we focus on the sparse representation approach, we explore the state-of-the-art category. Due to the appeal of many researchers in using sparse representation, approximating data by considering a few dictionary atoms was proposed [31,32,33,34,35,36,37,38,39,40,41,42]. A relaxed collaborative representation (RCR) approach was proposed in [33]. They speculated that their coefficients represented different features, and thus obtained the result by minimizing the sparse codes by counting the sum of the distances of coefficients from their average. Yuan et al. in [34] considered the $l_{1},l_{2}$ norm to obtain a joint sparse representation for the multiple features (MTJSRC), and they also tested their methods on the data with high dimensionality. Li et al. [36] proposed a multi-view multi-instance learning algorithm that creates a cohesive framework by incorporating several inner contextual structures from diverse perspectives.

Reference [38] presented a joint feature extraction to align multifeatures group and introduces a feature selection method for dimensionality reduction. Partial multiview clustering (PVC) was presented in [41], in which data were considered with an incomplete view. They used non-negative matrix factorization (NMF) [42] to train a latent subspace. In [31,39], a sparse representation model based on dictionary learning was introduced that obtained promising results when the multimodal features were considered. Due to assumption that there exist missed data in the multifeature extraction step, Zhao et al. [40] presented a partial multifeature unsupervised framework by preserving the similarity structure across different features. Nonparametric sparsity-based learning to reduce the dimensionality of multifeatures using the matrix decomposition method is presented in [37]. In [35], authors learned multifeatures extracted for diabetes mellitus and impaired glucose regulation problems using both specific and similar components, and then reported the effective results.

**Figure 2:**
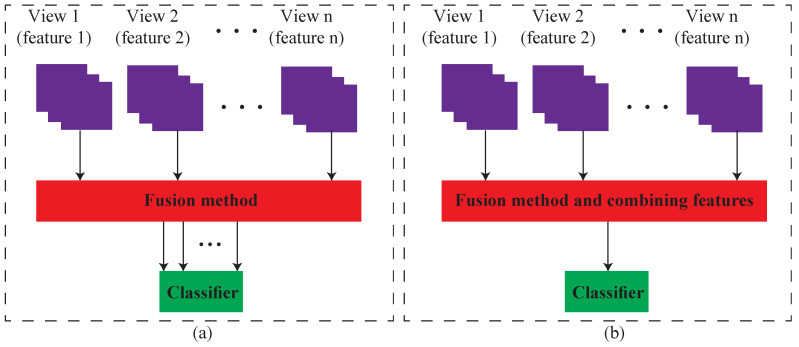
Different structures for multi-feature fusion. (**a**) multiple views are sent to classifier without reducing them (our approach) (**b**) Views are reduced into one map.

Although the mentioned methods to fuse multiple features achieved promising results in different classifications and clustering applications, the methods can be improved by some changes. To improve these methods, we propose a novel multifeature learning model. In general, the methods use all features simultaneously and follow two common structures, as shown in Figure 2.

## 3. Methodology

This section discusses applying the proposed method on the KERTAS database.

### 3.1. Database

**Figure 3:**
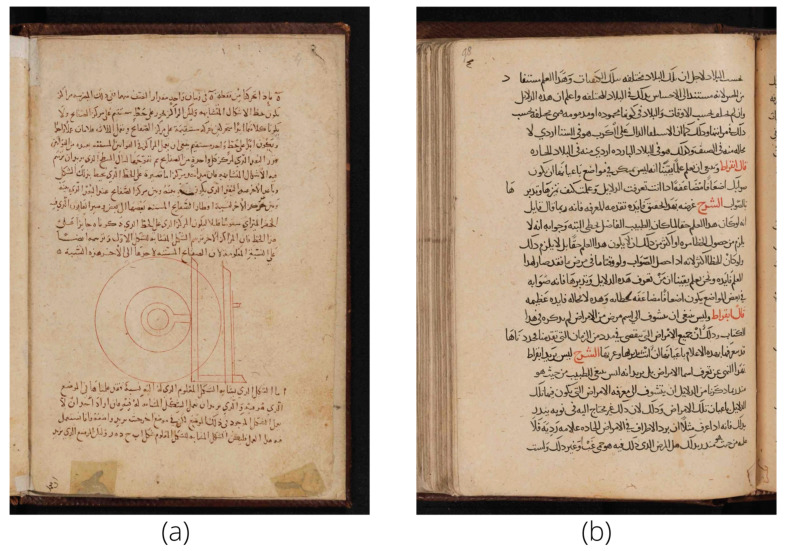
Samples of KERTAS dataset images (**a**) from 3rd Islamic century, and (**b**) from 7th Islamic century.

KERTAS dataset is a dataset for Historical Arabic Manuscripts, and it was first introduced in [16]. KERTAS dataset consists of over 2000 high-quality, high-resolution digital images acquired from the 1st to 14th AH century. Each class contains manuscripts from the same century; therefore, there are 14 classes in the database. A summary of the numerical distribution of documents in KERTAS and the number of images we used for training and testing are shown in Table 1. Additionally, two samples of the database are shown in Figure 3. For our experiment, we used 80% of the database for training and 20% for testing.

### 3.2. Preprocessing and Feature Extraction Methods

We started by segmenting the text area in the manuscript image to eliminate extra noise around the text. Afterward, we extracted features using the Gobor, edge hinge HOG, and ResNet methods. The selected features are some of the state-of-the-art, writing-style-based features that were used in multiple studies [43,44,45,46,47].

The Gabor filter is a feature descriptor used for texture and pattern detection comparable to the human visual system. A Gabor filter is modulated by a 2D-Gaussian function that can be viewed as a specific frequency and orientation sinusoidal plane. Gabor filters were used as a powerful feature to identify Arabic handwritten characters and words in several studies, as in [48,49,50]. The Histogram of Oriented Gradient (HOG) was initially introduced by [47] for face and human body detection. HOG is intended to define the structural shape of objects based on the distribution of directions and gradients of edges. The technique segments images of objects into smaller regions and then computes the histogram of gradient and edge directions based on the central differences. The histogram of oriented gradient was considered as a feature to capture the difference in letter representation due to changes in the style of handwriting and writing tools. Early styles tended to have thicker writing with rougher edges than that of later scripts. The edge-hinge is obtained by calculating the normalized histogram of the curvature edge of the text.The edge-hinge was used to identify writing styles, such as [43,45,46].

Lastly, a transfer learning method with a deep residual network or ResNet [51] was used to extract deep features that are added to the hierarchical fusion. We adopt ResNet with 18 layers deep in this research.

### 3.3. Hierarchical Fusion Approach

One of the efficient tools for fusing multifeatures is joint sparse representation [52,53]. If we have FE=[1,…,FE] as a finite set of available feature extraction methods and $X^{FE}=\left[x_{1}^{fe},x_{2}^{fe},\ldots ,x_{N}^{fe}\right]\in R^{n^{fe}\times N},fe\in FE$ as the collection of *N* (normalized) training samples of the methods, we can assume independence of the data statistically ($x^{fe}$ is the feature vector for the sth method). To address fusion step, the method formulates it by dictionary representation $D^{fe}\in R^{n^{fe}\times d}$ the corresponding for the sth method. Therefore, we have the multifeature dictionaries constructed by data extracted from different methods. That is, jth atom of dictionary $D^{fe}$ is the jth data produced by the feth method. If $\left\{x^{fe}|fe\in FE\right\}$ be the sample of multifeature, we can solve the $l_{12}$-regularized reconstruction problem to obtain optimal code sparse matrix $A^{*}\in R^{d\times FE}$:

(1) $$\begin{array}{c} \underset{A\left[\alpha^{1}\ldots \alpha^{FE}\right]}{arg min}\frac{1}{2}\sum_{fe=1}^{FE}{∥x^{fe}-D^{fe}\alpha^{fe}∥}_{l_{2}}^{2}+\lambda {∥A∥}_{l_{12}}, \end{array}$$

where the regularizing parameter is λ. Here $\alpha^{fe}$ is the feth- column of *A* which shows the sparse representation for the feth method. The $l_{2}$ norm of a vector $x\in R^{m}$ and the $l_{12}$ norm of matrix $X\in R^{m\times n}$ are defined as ${∥x∥}_{l2}={\left(\sum_{j=1}^{m}{\left|x_{j}\right|}^{2}\right)}^{1/2}$ and $∥X∥l_{12}=\sum_{i=1}^{m}{∥x_{i\to }∥}_{l_{2}}$ ($x_{i\to }$ is the ith row of matrix), respectively. To solve the optimization problem, several algorithms were proposed [54], and to find $A^{*}$, we apply the efficient method of multipliers (ADMM) [55]. In addition, to obtain dictionaries, we apply the dictionary learning method based on multifeatures presented in [31].

To implement our approach based on the fusion method, we define set of $FE_{l_{i}}=\left[FE_{l_{0}},FE_{l_{1}},\ldots ,FE_{l_{n}}\right]$, in which *l* shows the level of features extracted and *i* depends on type of selecting subsets, e.g., $FE_{l_{0}}$ and $FE_{l_{1}}$ are raw features (zero level) and output of the fusion method in the first level. Features extracted in each level are defined as $X^{FE_{l_{ij}}}$ where *i* and *j* show the level of feature (view) and number of features (view), e.g., $X^{FE_{l_{03}}}$ is the third feature (view) in zero level (raw features). Given $P_{l_{i}}\left(X^{FE_{l_{ij}}}\right)$ is the set of all subsets of $X^{FE_{l_{ij}}}$ except to *∅* and with members less than two members. Set of $S_{l_{i}}$ is one subset of $P_{l_{i}}\left(X^{FE_{l_{ij}}}\right)$. To obtain $P_{l_{i+1}}\left(X^{FE_{l_{(i+1)j}}}\right)$, we have the equation as follows:

(2) $$\begin{array}{c} P_{l_{i+1}}\left(X^{FE_{l_{(i+1)j}}}\right)=P_{l_{i}}\left(X^{FE_{l_{ij}}}\right)-S_{l_{i}}+X^{FE_{l_{(i+1)j}}} \end{array}$$

If the number of members of $S_{l_{0}}$ equals to the number of raw features, we obtain the results of Equation (1). In the addition, we summarize the steps to obtain the final features in Algorithm 1.
**Algorithm 1** Feature extraction algorithm based on hierarchical fusion approach.**Input:** Raw features (views) $X^{FE_{l_{0j}}}$, j=1,2,…,n, regularization parameter λ, i=0. **Output:** Fused features $X^{FE_{l_{ij}}}$.
1:Compute the set of all subsets of $X^{FE_{l_{ij}}}=X^{FE_{l_{0j}}}$ except to *∅* and with members less than two members: $P_{l_{i}}\left(X^{FE_{l_{ij}}}\right)$.2:**repeat**3:    Select one of the subset $P_{l_{i}}\left(X^{FE_{l_{ij}}}\right)$: $S_{l_{i}}$.4:    Compute dictionaries set of $S_{l_{i}}$ using [31].5:    i=i+1.6:    Apply fusion method using (1): $X^{FE_{l_{ij}}}$.7:    Compute updated set of $P_{l_{i}}\left(X^{FE_{l_{(i)j}}}\right)$ using (2).8:**until** ($P_{l_{i}}\left(X^{FE_{l_{(i-1)j}}}\right)-S_{l_{i-1}}\neq \emptyset$)


### 3.4. Classification

For classification of handwritten documents into year classes and to provide a fair comparison, we apply classifier used in [31]. The classifier is based on the joint sparsity prior to enforce collaborations among the multifeatures and obtain the latent sparse codes as the optimized features for multiclass classification. We present the performance of these classifiers in the next section. To make the final decision of the classifiers, there are several ways to do so, such as adding corresponding scores and majority voting. In the study, the sum of the score for each feature group is used.

## 4. Experimental Results

To evaluate the efficacy of the proposed system, experiments are conducted on the KERTAS dataset, and the described method is also compared with the state of the art methods. The experiments are elaborated in detail in the next subsections.

The performance of the method is measured by computing the accuracy (%). Moreover, the problem of dating manuscripts is usually evaluated by the mean absolute error (MAE). The calculation of the MAE is summarized in Equation (3) [18], where $K\bar{(}y_{i})$ is the true year of the input document $y_{i}$, $K(y_{i})$ is the estimated year, and *N* is the number of test documents. A lower value of MAE indicates better system performance:

(3) $$\begin{array}{c} MAE=\sum_{i=1}^{N}\left|K\bar{(}y_{i})-K\left(y_{i}\right)\right|/N \end{array}$$

### 4.1. Setting

As mentioned in Section 3.1, we used the KERTAS dataset that is included with different years classes. We performed all simulations in MATLAB R2019a. All experiments are run on a 64-bit operating system with a CPU E5-2690 v3 @ 2.60 GHz, 64.0 GB of RAM. In the joint sparse representation, regularization parameters $\lambda_{1}$ are selected using cross-validation in the sets $\left\{0.01+0.005t|t\in \left\{-3,3\right\}\right\}$. The parameter $\lambda_{2}$ is set to zero in most of the experiments, as proposed in [31].

### 4.2. Results

The proposed method is compared with the other applied approaches that were applied on the KERTAS dataset as per the literature. The performance evaluation results on the dataset for the different features and our fusion approach are summarized in Table 2 for both supervised and unsupervised classifiers. The table shows that our approach achieves the best result in terms of accuracy and MAE compared to the results of the individual features and the concatenated features.

**Figure 4:**
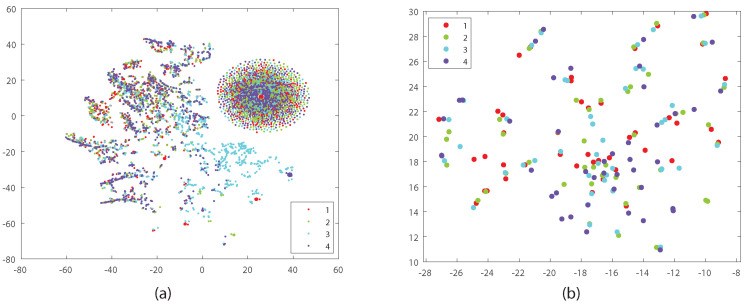
(**a**) concatenated original data; (**b**) our approach using t-SNE on KERTAS dataset (based on four views).

To analyze the learned feature space, we used the t-SNE algorithm [56] with respect to the KERTAS dataset to project 10 samples of the first class onto the two dimensions, as shown in Figure 4. The samples are based on the four views. As shown in Figure 4a, the original data consist of two main parts in the feature space, while our proposed approach (Figure 4b) assigns the features to only one part, which leads the classifier to obtain more accurate results than the method based on concatenation of features.

In the next subsection, we explore the several setups for our proposed approach.

#### The Impact of Different Setups

**Figure 5:**
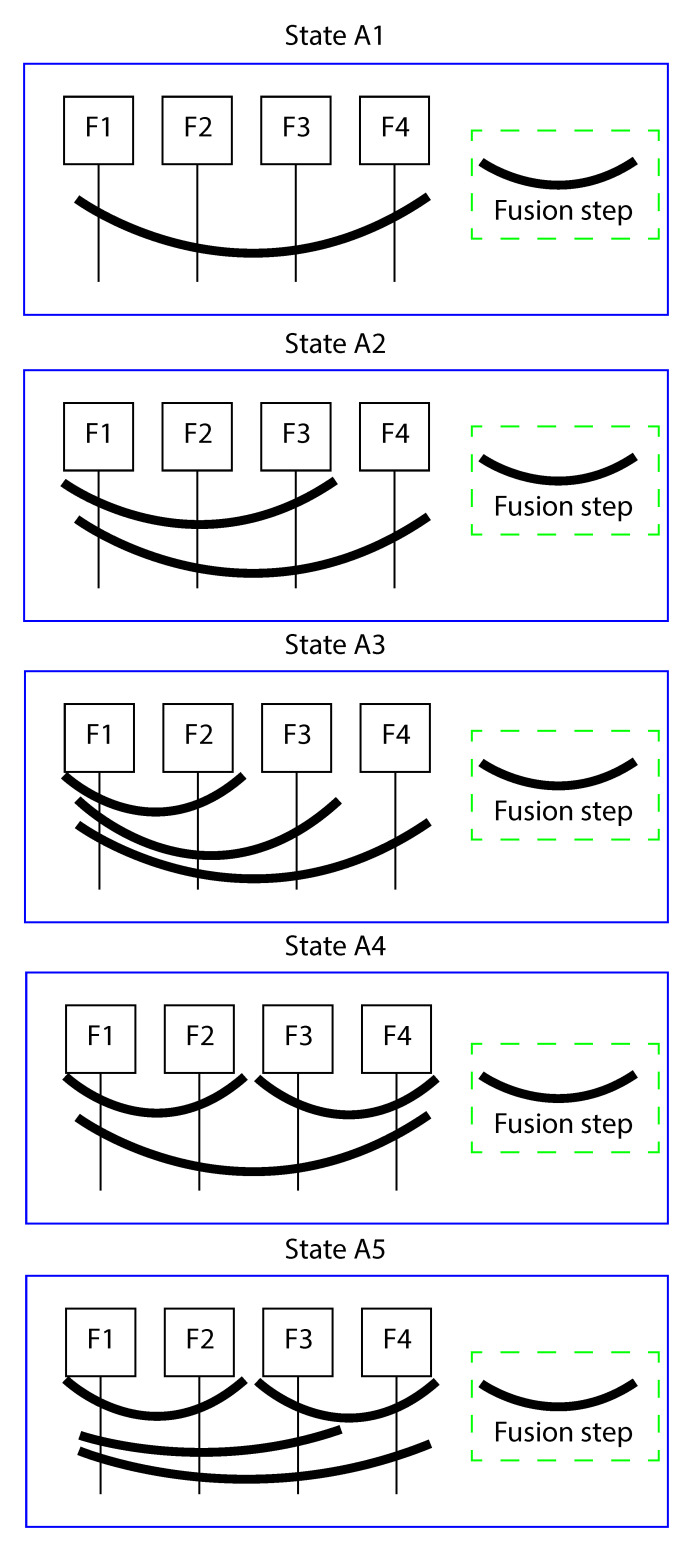
Different setups of our approach.

As shown in Figure 5, we consider five states based on our approach. The classification rates are computed and are illustrated in Table 3. The results show that all hierarchical states (states A2, A2, A4, and A5) obtain significant improvement in terms of classical state of fusion method (state A1 [31]). Also, when we use two subsets with size larger than two (state A5), we obtain the best result.

## 5. Conclusions

Automatic dating systems for historical manuscripts can considerably assist paleographers in obtaining better results with sufficient accuracy. Several dating methods were proposed for Arabic manuscript dating, but most of these methods need further improved outcomes. This paper presents a novel approach that improves classical dating methods by applying feature-level hierarchical fusion. Generally, features can have data with noise, which increases when more than one feature is applied. A new approach based on subsets of the multifeatures is considered to reduce the impact of fusion methods. In this study, we use traditional and deep convolutional neural network features applied to the manuscripts and introduced them as state-of-the-art features. We show that applying a hierarchical fusion based on subsets of multifeatures in the KERTAS dataset can obtain promising results and substantially improve the results as well.

In future work, our model will be customized to address the issues of multiclass classification in other applications. Additionally, we aim to develop the model to select subsets based on the best approach.

## Figures and Tables

**Table 1:** Summary of numerical distribution of documents in KERTAS dataset.

Key Century	Number of Documents	Training	Testing
1	60	48	12
2	47	37	10
3	144	116	28
4	592	474	118
5	164	132	32
6	119	95	24
7	184	147	37
8	110	88	22
9	153	123	30
10	73	59	14
11	169	135	34
12	147	118	29
13	119	95	24
14	17	14	3

**Table 2:** Results of different feature extraction methods and the best results of our fusion approach on KERTAS dataset (test set). (Best values are highlighted in bold).

Methods	Unsupervised MAE (%)	Accuracy (%)	Supervise MAE (%)	Accuracy (%)
Gabor	50.40	45.71	35.65	66.66
Hinge	49.21	47.61	37.31	61.90
Hog	52.80	43.80	37.35	61.90
ResNet	43.80	55.23	33.30	69.52
Concatenated	39.35	61.90	31.50	71.42
features				
Ours	**31.95**	**71.25**	**26.90**	**82.50**

**Table 3:** Comparison between different setups of our approach in terms of accuracy (%). (Best values highlighted in bold).

State	Unsupervised (%)	Supervise (%)
A1	64.28	75.47
A2	64.95	75.45
A3	67.65	76.85
A4	69.22	80.95
A5	**71.25**	**82.50**

## Data Availability

Not applicable.
